# A protocol for the management of pediatric type I open fractures

**DOI:** 10.1007/s11832-014-0554-7

**Published:** 2014-01-25

**Authors:** Christopher A. Iobst, Craig Spurdle, Avi C. Baitner, Wesley F. King, Michael Tidwell, Stephen Swirsky

**Affiliations:** Miami Children’s Hospital, 3100 SW 62nd Avenue, Miami, FL 33155 USA

**Keywords:** Open fracture, Pediatric

## Abstract

**Background:**

The management of pediatric type I open fractures remains controversial. There has been no consistent protocol established in the literature for the non-operative management of these injuries.

**Methods:**

A protocol was developed at our institution for the non-operative management of pediatric type I open forearm fractures. Each patient was given a dose of intravenous antibiotics at the time of the initial evaluation in the emergency department. The wound was then irrigated and a closed reduction performed in the emergency department. The patient was admitted for three doses of intravenous antibiotics (over approximately a 24-h period) and then discharged home without oral antibiotics.

**Results:**

In total, 45 consecutive patients were managed with this protocol at our hospital between 2004 and 2008. The average age was 10 (range 4–17) years. The average number of doses of intravenous antibiotics was 4.06 per patient. Thirty patients (67 %) received cefazolin (Ancef®) as the treating medication and 15 patients received clindamycin (33 %). There were no infections in any of the 45 patients.

**Conclusion:**

In this study we outline a consistent management protocol for type I open pediatric forearm fractures that has not previously been documented in the literature. Our results corroborate the those reported in the literature that pediatric type I open fractures may be managed safely in a non-operative manner. There were no infections in our prospective series of 45 consecutive type I open pediatric forearm fractures using our protocol. Using a protocol of only four doses of intravenous antibiotics (one in the emergency department and three additional doses during a 24-h hospital admission) is a safe and efficient method for managing routine pediatric type I open fractures non-operatively.

## Introduction

The management of pediatric type I open fractures remains controversial. The traditional approach involves taking the patient to the operating room in an urgent time frame for irrigation and debridement of the open wound and bone ends through an extensile incision. This method is still recommended in most orthopedic textbooks and review articles [1–4]. However, it has recently been reported in a number of publications that operative management may not always be necessary for pediatric type I open fractures [5–7].

Regardless of whether an operative or non-operative approach is chosen, prompt administration of intravenous antibiotics is an important element in the management of open fractures. Patzakis and Wilkins [8] reported an infection rate of 4.7 % in patients in whom antibiotics had been administered within 3 h after the injury and 7.4 % in patients who had received antibiotics more than 3 h after the injury. This is especially relevant in type I open fractures where a relatively intact soft tissue envelope allows penetration of the antibiotics to the area of injury.

The exact duration of antibiotic treatment necessary for effective management of a pediatric type I open fracture, however, has not been determined. Previous research on this topic demonstrated variability in both the choice of antibiotic and the number of doses given [6]. The new protocol which we describe herein is designed to standardize the non-operative management of each pediatric patient with a type I open forearm fractures by using specific antibiotics and a defined number of doses. According to this protocol, each patient in our study cohort was given a dose of intravenous antibiotics at the time of the initial evaluation in the emergency department. The wound was then irrigated and a closed reduction performed in the emergency department, following which the patient was admitted to hospital for three doses of intravenous antibiotics (over an approximately 24-h period) and then discharged home without oral antibiotics. Here, we present our review of our institution’s preliminary results on the use of this protocol in pediatric type I open forearm fractures.

## Materials and methods

**Fig. 1:**
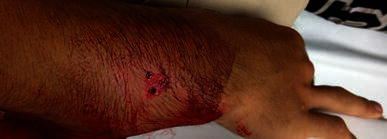
Nine-year-old male with type I open distal radius and ulna fracture after a fall on the trampoline. Clinical photo of open wound on dorsum of forearm from proximal spike of distal radius fracture fragment (inside–out wound)

After obtaining Institutional Review Board approval, we performed a prospective study on all pediatric type I open fracture patients presenting to our institution between 2004 and 2008. The protocol described herein was initiated at our institution in 2004 for the non-operative management of these injuries. If a pediatric orthopedic fracture patient in the emergency department is found to have an open wound, the wound is evaluated by the resident physician or physician assistant on call. The wound is then measured and inspected in the patient’s emergency department room with standard overhead lighting to determine if it is a superficial abrasion or a full thickness breach of the skin. If the wound is full thickness, then it is evaluated as to whether it communicates with the fracture site or if it is remote and an independent injury. All full thickness wounds of ≤1 cm in size that are suspicious for communication with the fracture site are classified as type I open fractures in accordance with the Gustilo and Anderson [9] classification and the patients are started on the protocol (see Fig. 1).

**Fig. 2:**
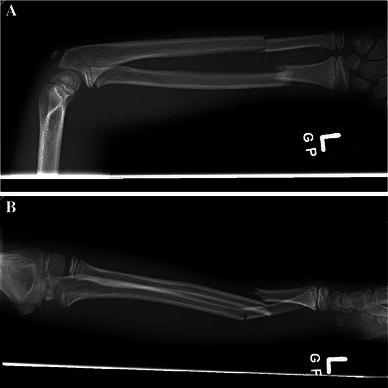
**a**, **b** Injury radiographs demonstrating displaced radius and ulna fractures

**Fig. 3:**
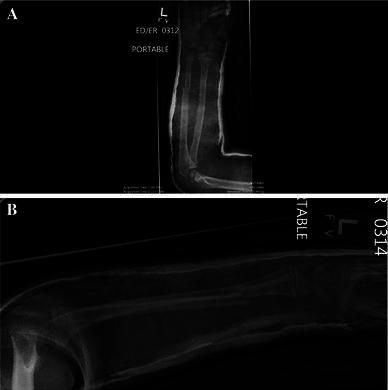
**a**, **b** Immediate post-reduction radiographs in the emergency department demonstrating satisfactory reduction of the fractures in a long arm cast

**Fig. 4:**
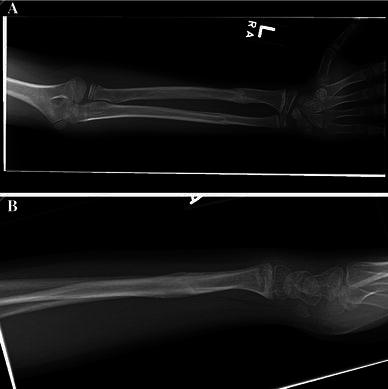
**a**, **b** Fracture at time of cast removal 52 days after injury

First, each patient identified in the emergency department as having a type I open fracture is given a weight appropriate dose of intravenous antibiotics as soon as possible. Next, the wound is irrigated at the bedside with a 1 l mixture of 1 % Betadine® and sterile saline solution. The wound is then dressed with Xeroform^TM^ gauze and a sterile gauze dressing. Closed reduction of the fracture is then performed, and the patient is immobilized in a cast or a splint depending of the amount of soft tissue swelling (see Figs. 2 and 3). If a cast is applied, the site of the wound is windowed to allow future visualization. The patient is subsequently admitted to the hospital for three more doses of intravenous antibiotics (over approximately 24-h period). No wound cultures are obtained. Prior to discharge the wound is inspected through the window in the cast or splint. Finally, the patient is discharged home without any further antibiotics (oral or intravenous) and is given an appointment to be seen in the office in 1 week. At the 1-week follow-up appointment, the wound is again inspected through the window in the cast. Radiographs for routine fracture care are obtained. Assuming there is no loss of reduction, the fracture is then seen every 2–3 weeks for routine follow-up until clinical healing has occurred (see Fig. 4).

All patients with type I open fractures seen in our emergency department between 2004 and 2008 were included in the study. A total of 49 consecutive patients were identified. Two patients were lost to follow-up. Each of these two patients was injured while on vacation in our area but returned to home after receiving initial management in our institution. Two fractures involved the tibia and were managed with the current protocol and healed with excellent results. However, since there were so few lower extremity fractures in the series, we did not feel any reliable conclusions could be ascertained. Therefore, the two tibia fractures were not included in the results. The remaining 45 patients were included in the study. Clinical and demographic data were recorded for each patient, including age, gender, bone fractured, site of fracture within each bone, mechanism of injury, type of antibiotic given, number of doses of antibiotics given, approximate time from injury to first dose of antibiotics, time from emergency department triage to first dose of antibiotics, whether the patient was transferred from another medical facility, time to transfer patient from other medical facility, length of hospital admission, number of follow-up visits, time to healing of fracture, and whether any operative intervention was required during the course of fracture management. Complications were also recorded, including loss of reduction, infection, and re-fractures after cast removal.

## Results

Forty-five consecutive pediatric type I open forearm fractures were managed with the outlined protocol. The clinical and demographic data on these pediatric patients are summarized in Table 1. The average age of these patients was 10 (range 4–17) years, and there were 36 boys and nine girls. Nineteen fractures involved the right extremity and 26 involved the left extremity. The forearm fractures were located in the proximal third in three patients (7 %), the middle third in 17 patients (38 %) and the distal third in 25 patients (55 %). The mechanism of injury for the 45 patients included fall from height (fence, monkeybars, tree, bed, truck, countertop, table, wheelchair; 9 patients); fall from standing height (9 patients); sports injury (9 patients); fall off bicycle (6 patients); fall off skateboard (6 patients); fall skating (5 patients); hit by car (1 patient). The average estimated time from injury to the first dose of antibiotics was 4 h and 52 min (range 67–600 min). The average time from triage to first dose of antibiotics was 4 h and 4 min (range 15–513 min). Fourteen patients (31 %) were transferred from another emergency department to our institution for definitive care. The average time to arrange the transfer was 3 h and 52 min (range 160–366 min).

**Table 1:** Clinical and demographic data on the 45 pediatric patients with type I open forearm fractures managed with the outlined protocol

Clinical and demographic parameters	Values
Average age (years)	10 (range 4–17)
Gender (male:female)	36 (80 %):9 (20 %)
Fracture side (right vs. left)	19 (42 %) vs. 26 (58 %)
Fracture location in forearm	Proximal (3 patients, 7 %) Middle (7 patients, 38 %) Distal (25 patients, 55 %)
Estimated time from injury to first dose of antibiotics	4 h 52 min (range 67–600 min)
Time from triage to first dose of antibiotics	4 h 4 min (range 15–513 min)
Average number of antibiotic doses	4.06
Average length of admission	41 h 27 min (range 26 h 37 min to 64 h 8 min)
Average healing time for fracture	50.5 (range 32–84) days
Infections	0

The average number of doses of intravenous antibiotics was 4.06 per patient. Thirty patients (64 %) received Ancef® (cefazolin)as the treating medication and 15 patients received clindamycin (36 %). The average dose of Ancef® and clindamycin was 21.7 (range 11.9–45) mg/kg and 12.1 (range 9.8–15.2) mg/kg, respectively. The average length of admission was 41 h and 27 min (range 26 h 37 min to 64 h 8 min). The patients had an average of 3.44 follow-up visits. The average healing time for the forearm fractures was 50.5 (range 32–84) days. All patients achieved healing of the fracture with satisfactory alignment at the completion of treatment. However, three patients (6 %) required operative intervention for the loss of reduction in the cast (two closed reduction with pins and one repeat closed reduction). There were two re-fractures after cast removal (4 %). Both patients went on to satisfactory healing with re-immobilization. There were no infections in any of the 45 patients. A period of between 5 and 9 years has now passed since the acute fracture care for this series of patients ended. During that time span no patient from this series has returned to the emergency department or orthopedic surgery department with an infection.

## Discussion

The management of type I open fractures has traditionally been operative. Most orthopedic textbooks describe taking patients with these injuries to the operating room in an urgent or emergent fashion for the purposes of debriding the wound through an extensile incision [1–4]. However, since routine type I open fractures by definition have minimal soft tissue disruption, there is usually adequate vascularization to the area of injury [10]. This allows the patient’s immune response to have access to any contaminant bacteria. It also allows the delivery of antibiotics to the zone of injury. Therefore, theoretically, it can be argued that type I open fractures may be managed non-operatively with closed reduction and appropriate antibiotics.

There is no one specific intravenous antibiotic used to manage all type I open forearm fractures. Under the advice of our hospital infectious disease team, the orthopedic department switched our standard intravenous antibiotic of choice from Ancef® (cefazolin) to clindamycin to obtain better coverage against methicillin-resistant *Staphylococcus aureus* in all orthopedic patients. This recommendation occurred during the course of our study collection period and, consequently, the initial group of patients received Ancef® and the subsequent group of patients received clindamycin. This change does not reflect any failure of treatment with Ancef® but was only a change in hospital policy. We suggest that treating physicians consult their respective infectious disease department for the most appropriate antibiotic choice for their respective hospital. Regardless of the antibiotic chosen, we did not find any difference in the infection rate (Ancef® 0/30 patients = 0 %; clindamycin 0/15 patients = 0 %).

While the specific type of antibiotic chosen is certainly critical, it may be more important that it is administered in a timely fashion. Experimental results reported in the literature demonstrate that the infection rate of open fractures decreases when antibiotics are given before debridement [8]. It is recommended that the intravenous antibiotics should be given as soon as possible, preferably within 3 h after the injury [8]. In our series, the antibiotics were administered within an average of 4 h and 4 min from the time of triage in the emergency department. As a result of this investigation, we are working with our emergency department staff to improve the timing of the initial antibiotic dose.

The mechanisms of injury indicate that the type I open wounds were created in a variety of different environments. Ten of the wounds (22 %) occurred inside the home and 35 occurred while the patient was performing activities outside the home (78 %). The open fractures that happened outside the home could be viewed as potentially “dirtier” wounds, but there was no difference in the infection rate between the two groups (0 %). This series, however, contained no grossly contaminated barnyard wounds. The authors recommend using clinical judgment on a case by case basis regarding whether or not surgical debridement is necessary.

The reliability of the Gustilo and Anderson classification system has been questioned in the literature [11]. Given that our evaluation of each patient’s wound was done in the emergency department at the bedside without the assistance of operating room lighting and surgical equipment, it is possible that some of our type I open fractures may have actually been type II open fractures. Nevertheless, the treatment protocol was successful in managing each of these injuries even if a few were incorrectly labeled as a type I open fracture. This investigation does not advocate routine non-operative management for type II open fractures. However, based on the results of this series and its inherent possible classification errors, fractures on the borderline between type I and type II may not need surgical debridement in all cases.

Recent studies have demonstrated that non-operative management of type I open fractures may be safe and efficacious. In 2003 Yang and Eisler reported a 0 % infection rate in 90 type I open fracture patients, even though none of the patients were taken to the operating room within 8 h and only 32 of the patients were taken to the operating room at all. Each patient received 48 h of antibiotics and 13 of the patients were children [5]. In 2005, Iobst et al. [6] reviewed 40 pediatric patients with type I open fractures managed non-operatively and found one patient that developed a late infection (2.5 %). These patients were hospitalized for an average of 2.5 days and received an average of 6.97 doses of intravenous antibiotics [6]. In 2009, Doak and Ferrick [7] reported one infection out of 25 pediatric type I open fractures (4 %) managed non-operatively. Each patient was given intravenous antibiotics in the emergency department and over the course of the hospital admission, but no patient was admitted for >24 h. At discharge, 80 % of the patients were receiving oral antibiotics with the duration of treatment ranging from one to seven additional days after discharge.

The results of our current study corroborate those previously reported in literature that pediatric type I open fractures may be managed safely in a non-operative manner. There were no infections in our series of 45 consecutive type I open pediatric forearm fractures using our current protocol. There was also no significant delay in the healing of the fractures, with an average healing time of 7 weeks for the 45 forearm fractures. Combining the results of this series with the other three published studies on this subject reveals an infection rate of 1 % (2/200) for type I open fractures managed non-operatively [5–7]. This compares favorably with the infection rate reported in the literature (1.9 %) for type I open fractures managed operatively [6, 12].

This study attempts to provide a clear and coherent protocol for the non-operative management of pediatric type I open forearm fractures. Previous literature on this subject has been inconsistent in the recommended antibiotic treatment [5–7]. The described duration of intravenous antibiotics ranges from one dose to seven doses. There is also disagreement, even within a study, about whether oral antibiotics are necessary after discharge. In our protocol, each patient was given a dose of intravenous antibiotics (Ancef® or clindamycin) as soon as possible in the emergency department. Three additional doses were given over the course of a 24-h period during hospital admission. During the 24 h admission, the extremity was monitored for signs and symptoms of a compartment syndrome and the wound was inspected again prior to discharge. Each patient was discharged to home without any oral antibiotics. While Doak and Ferrick’s [7] idea to send the patients home on oral antibiotics after a single dose of intravenous antibiotics in the emergency room is admirable, in our patient population it would be risky to assume that the families will actually be compliant with giving oral antibiotics as prescribed even for a short period of time. Consequently, we feel safer admitting the patients to ensure that a complete course of antibiotics is properly administered and received.

## Conclusion

Based on our preliminary results, it appears that a protocol using four doses of intravenous antibiotics (one in the emergency department and three additional doses during a 24-h period of hospital admission) is a safe and efficient method for managing routine pediatric type I open forearm fractures non-operatively. However, the use of antibiotics is not advocated as a substitute for proper clinical judgment, and the decision to treat pediatric type I open fractures non-operatively should continue to be made on a case by case basis. Pediatric type I open fractures of the lower extremity may react differently and need further study before any recommendations can be made regarding treatment. For now, based on our experience with this protocol, we limit our recommendations for non-operative management only to pediatric type I open forearm fractures.

## References

[1] Price CT, Flynn JM, Morrissy RT, Weinstein SL (2006). Management of fractures. Pediatric orthopaedics.

[2] Herring JA, Herring JA (2008). General principles of management of orthopedic injuries. Pediatric orthopaedics.

[3] Tolo VT, Beaty JH, Kasser JR (2001). Management of the multiply injured child. Fractures in children.

[4] Stewart DG, Kay RM, Skaggs DL (2005). Open fractures in children. J Bone Joint Surg.

[5] Yang EC, Eisler J (2003). Treatment of isolated type I open fractures: is emergent operative debridement necessary?. Clin Orthop.

[6] Iobst CA, Tidwell MA, King WF (2005). Nonoperative management of pediatric type I open fractures. J Pediatr Orthop.

[7] Doak J, Ferrick M (2009). Nonoperative management of pediatric grade 1 open fractures with less than a 24-hour admission. J Pediatr Orthop.

[8] Patzakis MJ, Wilkins J (1989). Factors influencing infection rate in open fracture wounds. Clin Orthop.

[9] Gustilo RB, Anderson JT (1976). Prevention of infection in the treatment of one thousand and twenty-five open fractures of long bones. J Bone Joint Surg Am.

[10] Gustilo RB, Merkow RL, Templeman D (1990). The management of open fractures. J Bone Joint Surg.

[11] Brumback RJ, Jones AL (1994). Interobserver agreement in the classification of open fractures of the tibia. The results of a survey of two hundred and forty-five orthopaedic surgeons. J Bone Joint Surg Am.

[12] Lim YJ, Lam KS, Lee EH (2007). Open Gustilo 1 and 2 midshaft fractures o the radius and ulna in children. J Pediatr Orthop.

